# Probiotic Supplementation and Executive Function in Children With Attention‐Deficit/Hyperactivity Disorder

**DOI:** 10.1002/npr2.70084

**Published:** 2025-12-25

**Authors:** Abdolrahman Parhiz, Pegah Samani, Majid Kamali, Yeganeh Shekari, Niayesh Naghshi, Newsha Faghihshojaei, Faezeh Tejareh, Niloufar Pourmalek Lahiji, Ali Nori, Atefeh Aminifard, Parsa Bahmani, Saeid Doaei, Fatemeh Rabipour, Maryam Gholamalizadeh

**Affiliations:** ^1^ Kish International Campus University of Tehran Tehran Iran; ^2^ Nutrition and Food Security Research Center Shahid Sadoughi University of Medical Science Yazd Iran; ^3^ School of Nutrition and Food Sciences Isfahan University of Medical Sciences Isfahan Iran; ^4^ Department of Nutrition, Faculty of Medical Sciences Islamic Azad University Iran; ^5^ Departemen of Clinical Nutrition Shahid Beheshti University of Medical Sciences Tehran Iran; ^6^ Department of microbiology Lahijan Branch, Islamic Azad University Lahijan Iran; ^7^ Department of Nutrition Science and Research Branch, Islamic Azad university Tehran Iran; ^8^ Faculty of Sciences Guilan University Rasht Iran; ^9^ Department of Clinical Nutrition, School of Nutritional Sciences and Dietetics Tehran University of Medical Sciences Tehran Iran; ^10^ Department of Food Industry, School of Food Sciences and Industry Islamic Azad University Ahvaz Iran; ^11^ Department of Clinical Nutrition and Dietetics, Faculty of Nutrition and Food Technology Shahid Beheshti University of Medical Sciences Tehran Iran; ^12^ Reproductive Health Research Center, Department of Obstetrics & Gynecology, School of Medicine, Al‐Zahra Hospital Guilan University of Medical Sciences Rasht Iran; ^13^ Departemen of Psychology, Ra.C. Islamic Azad University Rasht Iran; ^14^ National Nutrition and Food Technology Research Institute, Faculty of Nutrition Sciences and Food Technology Shahid Beheshti University of Medical Sciences Tehran Iran

**Keywords:** attention‐deficit hyperactivity disorder (ADHD), executive function, probiotic

## Abstract

**Background:**

Attention Deficit Hyperactivity Disorder (ADHD) is a prevalent neurodevelopmental condition marked by inattention, hyperactivity, and impulsivity, which substantially impacts children's academic and social performance. The aim of this research was to examine the effect of probiotic supplementation on executive functioning in children with ADHD.

**Methods:**

This triple‐blind, randomized controlled trial included 84 children with ADHD who were randomly allocated to receive either a probiotic supplement including *Lactobacillus Acidophilus* , *Bifidobacterium Lactis* , and *Bifidobacterium Longum* or a placebo, administered once daily for 2 months. Executive function was assessed using the parent form of the Behavior Rating Inventory of Executive Function (BRIEF) questionnaire. The data were analyzed using repeated measures analysis of variance after adjusting the confounding variables.

**Results:**

No significant difference was found between executive function scores of the groups at baseline (191.45 ± 20.725 vs. 190.55 ± 23.520, *p* = 0.214). Probiotic treatment significantly improved the scores of the intervention group compared to the control group (151.50 ± 16.784 vs. 190.68 ± 23.479, *F* = 7.93, *p* < 0.001) after adjusting for the confounders including age, BMI, and IQ.

**Conclusion:**

Probiotics may be beneficial as non‐pharmacological adjunctive therapy for enhancing executive function in children with ADHD. Further studies with extended follow‐up durations and objective neuropsychological evaluations are warranted.

**Trial Registration:**

IRCT: IRCT20210531051454N2

## Abstract 2

Probiotics may be beneficial as nonpharmacological adjunctive therapy for enhancing executive function in children with ADHD.

## Graphical abstract

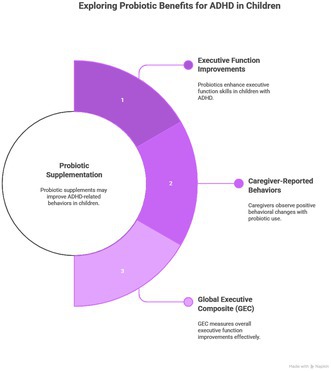

## 1 Introduction

Attention‐deficit hyperactivity disorder (ADHD) is a common neurodevelopmental disorder characterized by persistent patterns of inattention, hyperactivity, and impulsivity, which can considerably affect academic, social, and daily functioning [1]. The global prevalence of ADHD among children and adolescents is estimated to be 5.29%, making it one of the most prevalent psychiatric disorders in this age group [2, 3]. In Iran, the prevalence of ADHD has been reported at 22.2%, whereas the pooled prevalence across the Middle East and North Africa (MENA) region is estimated at 10.3%, with considerable variation between countries [4]. The management of ADHD typically involves a combination of pharmacological treatments, behavioral therapies, and educational interventions. However, these approaches often come with limitations, such as potential side effects of medications and the need for sustained behavioral interventions [5]. Consequently, there is an increasing focus on investigating alternative and adjunctive therapies that can mitigate symptoms and enhance cognitive functioning in children with ADHD [6, 7].

One emerging area of interest is the role of gut microbiota in brain function and behavior [8, 9]. The gut–brain axis represents a bidirectional communication network connecting the enteric and central nervous systems, highlighting the role of the gut microbiome in influencing brain activity, behavior, and cognitive functions [8]. Probiotics are live microorganisms that provide health benefits to the host when taken in sufficient quantities and have attracted interest for their potential positive effects on the gut‐brain axis [10]. Several studies suggested that probiotics may have a range of beneficial effects, including the modulation of immune responses, enhancement of gut barrier function, and production of neuroactive compounds [8, 10, 11].

In the context of ADHD, preliminary research indicated that probiotic supplementation may offer promising benefits [12]. Following probiotic administration, some studies observed improvements in behavioral symptoms, such as reduced hyperactivity and improved attention [12, 13]. However, the impact of probiotics on specific cognitive domains, particularly executive functions, remains underexplored. The Global Executive Composite (GEC) is a comprehensive metric obtained from the Behavior Rating Inventory of Executive Function (BRIEF). The GEC is considered a primary measure due to its comprehensive assessment of executive function impairments, which are commonly affected in ADHD, encompassing cognitive processes such as working memory, flexible thinking, and self‐control, which are essential for goal‐directed behavior and often impaired in children with ADHD [14]. The present study establishes a basis for examining the potential effects of probiotic supplementation in children diagnosed with ADHD. This study aims to address a gap in the literature by evaluating the effects of probiotic supplementation on executive function in children with ADHD. The findings may contribute to the development of novel nonpharmacological interventions that enhance cognitive function and quality of life.

## 2 Methods

### 2.1 Study Design and Implementation

This was a triple‐blind randomized controlled trial, in which patients, researchers, and statistical analysts were unaware of group allocation. Participants were selected from the individuals referred to the welfare organization, Shafa Hospital, and Specialized Centers for Psychiatry and Psychology in Rasht, Iran, in 2023. Participants were selected after an interview by a psychiatrist and on the basis of the inclusion and exclusion criteria. After gender matching using an online randomization tool (www.randomizer.org), 44 people were assigned to the intervention group and 44 to the placebo group. Allocation concealment was ensured using Sequentially Numbered, Opaque Sealed Envelopes (SNOSE). The sample size was calculated using the Openepi online software, taking into account an alpha of 0.05, a beta of 20%, and a 1:1 ratio between the intervention and control groups, including 36 people in each group. However, we increased the required sample size to 44 participants considering a possible 20% dropout. Therefore, 84 children with ADHD were assigned to the intervention and control groups. The intervention group received daily oral probiotic supplements, and the control group received a placebo with a supplement dose similar to that of the intervention group.

### 2.2 Inclusion and Exclusion Criteria of the Study

Inclusion criteria were as follows: a confirmed diagnosis of ADHD based on DSM‐5 criteria by a board‐certified psychiatrist (with type and severity assessed at baseline), age between 7 and 12 years, willingness to participate in the study, normal IQ (above 85) as measured by the Wechsler Intelligence Scale for Children, stable treatment with methylphenidate (10–30 mg/day, immediate or extended‐release) for at least 2 months prior to and during the study period without any dosage or formulation changes, and a body mass index (BMI) below +2 standard deviations. To assess ADHD symptom severity, the Swanson, Nolan, and Pelham Rating Scale (SNAP‐IV) was used. This 18‐item scale includes nine items for inattention, six for hyperactivity, and three for impulsivity, originally derived from DSM‐IV criteria [15]. Although based on DSM‐IV, the SNAP‐IV remains a widely used and validated tool for evaluating ADHD symptoms in both clinical and research settings, including those aligned with DSM‐5. Its psychometric validity has been previously confirmed in the Iranian population [16].

Exclusion criteria included comorbidity with other mental disorders such as autism spectrum disorder (ASD), mood disorders, anxiety disorders, tic disorders, and learning disabilities; presence of physical illnesses; concurrent use of other probiotic‐enriched foods or supplements during the study period; lack of willingness or cooperation or any complications affecting participation; and occurrence of events such as accidents, death, or other factors that could influence study variables (*n* = 4). No concurrent behavioral therapy or other interventions were introduced or altered during the trial.

### 2.3 Probiotic Supplementation

After obtaining written informed consent from all participants' legal guardians or parents, the questionnaires were completed by the same caregivers. The intervention group received a probiotic capsule (Bioflora, manufactured by Takgene Zist Pharmaceutical Company, Iran) once daily for 2 months. Each capsule contained *Lactobacillus acidophilus* , *Bifidobacterium lactis* , and *Bifidobacterium longum* , selected for their documented anti‐inflammatory, immunomodulatory, and gut–brain axis–modulating properties, along with excipients magnesium stearate and maltodextrin. The control group received a placebo capsule with identical excipients and appearance, also administered once daily for 2 months.

In order to follow up with the study participants, improve study compliance, and check the gastrointestinal symptoms or side effects in the participants, patients were contacted regularly (once a week) and in case of any problems, necessary actions and follow‐ups were taken by the project researchers. The research psychologist engaged with the children's guardians regarding the potential advantages of the study and urged them to persist with their participation. To facilitate adherence monitoring, the supplements were delivered to participants' homes in three separate batches of 20. Caregivers were instructed to return the previously used envelopes, allowing researchers to estimate compliance based on returned packaging.

### 2.4 Data Collection

Information on executive functions of the participants was collected during the pretest and posttest phases using the Behavior Rating Inventory of Executive Function (BRIEF—Parent Form), which has been validated in Iran [17]. This questionnaire consists of 86 items rated by parents on a 3‐point Likert scale (“never,” “sometimes,” and “often”) and is designed to assess executive functioning in children aged 5–18 years. For the purpose of this study, we focused specifically on the Global Executive Composite (GEC) score, which provides an overall index of executive functioning difficulties. Demographic and clinical information—including age, gender, parental education, residence, income, treatment history, and comorbid conditions—was collected via a standardized diagnostic interview form [17].

### 2.5 Statistical Analysis

Data analysis was performed using SPSS software (version 24) and included both descriptive and inferential components. Descriptive statistics, including measures of central tendency and dispersion such as mean and standard deviation, were used to summarize the baseline characteristics and outcome variables. To assess the normality of the data distribution, the Shapiro–Wilk test was applied. Upon confirming the assumption of normality, repeated measures analysis of variance (RM‐ANOVA) was employed to evaluate the study hypotheses and examine changes across the pre‐test and post‐test phases. In cases where the sphericity assumption was violated, Greenhouse–Geisser correction was applied to adjust the degrees of freedom. Additionally, effect sizes (partial eta squared) were reported to determine the magnitude of observed effects. All statistical analyses were performed using a significance threshold of *p* < 0.05.

## 3 Results

At baseline, no statistically significant difference was observed between the intervention and the control groups regarding age (9.48 ± 1.31 vs. 9.53 ± 1.33 years), gender distribution, IQ scores (98.59 ± 10.23 vs. 98.08 ± 8.58), body mass index (19.16 ± 1.75 vs. 19.23 ± 1.84 kg/m^2^), and Global Executive Composite (GEC) scores (191.45 ± 20.725 vs. 190.55 ± 23.520) (Table 1).

**TABLE 1:** General characteristics of the participants at baseline.

	Control (*n* = 40)	Intervention (*n* = 44)	*p*
Age (years)	9.48 ± 1.31	9.53 ± 1.33	0.877
Gender			
Male	27	31	0.816
Female	13	13	
IQ	98.08 ± 8.58	98.59 ± 10.23	0.804
BMI (kg/m^2^)	19.16 ± 1.75	19.23 ± 1.84	0.866
GEC	191.45 ± 20.725	190.55 ± 23.520	0.214

*Note:* One Sample *t*‐test and chi‐squared test were performed to calculate the significances.

Abbreviation: GEC, global executive composite.

**FIGURE 1:**
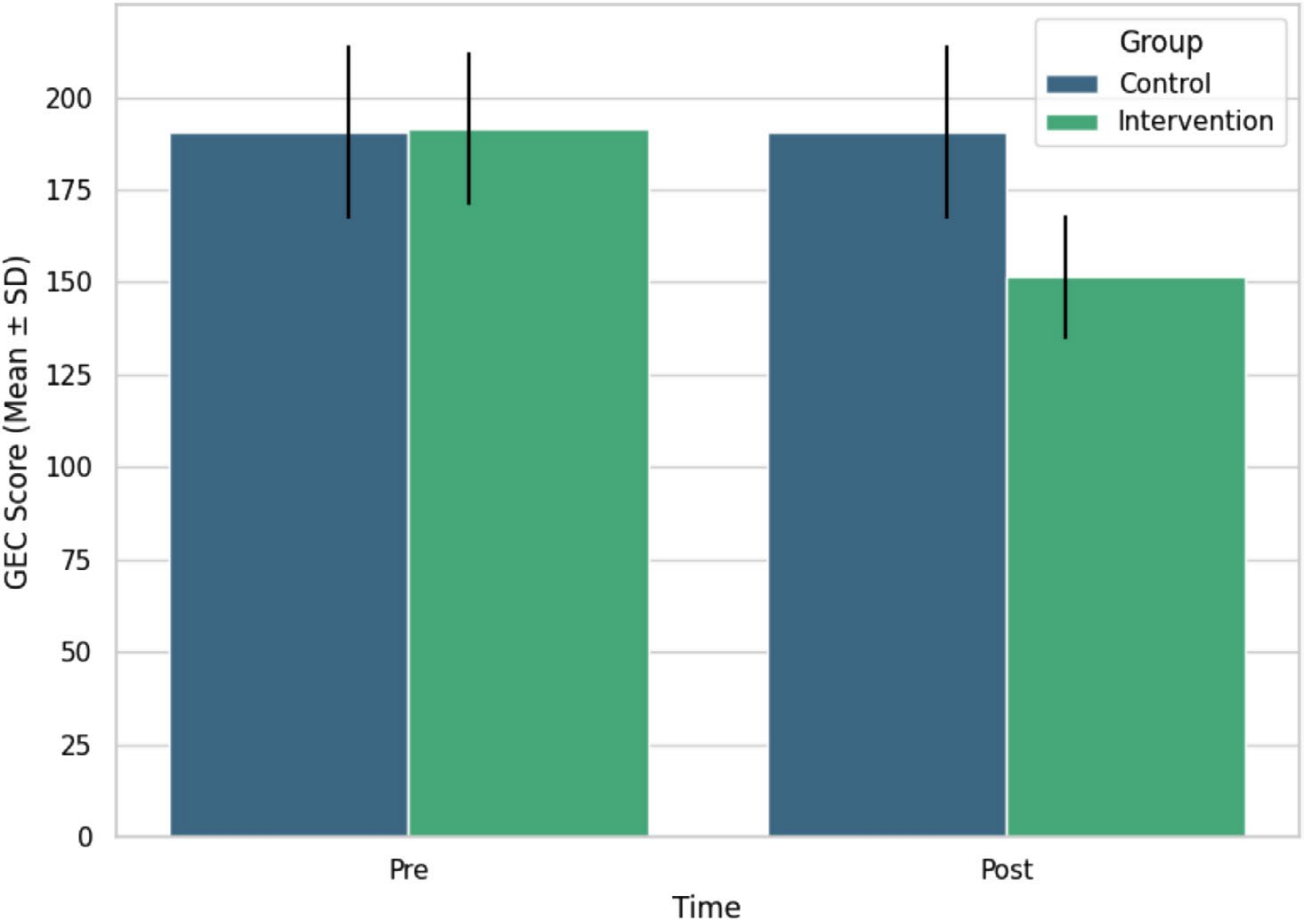
Effect of probiotic supplementation on GEC (global executive composite).

Regarding the effect of probiotic supplementation on GEC, following 2 months of the intervention, the probiotic group exhibited a significant enhancement in GEC scores relative to the control group (Figure 1). The probiotic group exhibited a substantial reduction in GEC scores, decreasing from 191.45 ± 20.725 at baseline to 151.50 ± 16.784 after the intervention (*F* = 7.93, *p* < 0.001). In the control group, GEC scores demonstrated little variation (190.55 ± 23.520 at baseline compared to 190.68 ± 23.479 postintervention) (Table 2).

**TABLE 2:** The effect of probiotic supplementation on GEC.

Factor	Intervention group means (SD)		Control group means (SD)		Group time	
	Pre	Post	Pre	Post	*F*	*p* ^a^
GEC	191.45 ± 20.725	151.50 ± 16.784	190.55 ± 23.520	190.68 ± 23.479	7.93	< 0.001

Abbreviation: GEC, global executive composite.

^a^ Adjusted for Age, BMI, and IQ.

## 4 Discussion

This study presents strong evidence supporting the potential advantages of probiotic supplementation in caregiver‐reported improvements in executive‐function‐related behaviors in children with ADHD, as assessed by the Global Executive Composite (GEC). Probiotic supplementation improved GEC scores compared to the control group, highlighting its possible role as a nonpharmacological adjunctive treatment. These results are consistent with prior studies emphasizing the impact of probiotics on neurodevelopmental and psychiatric conditions, including ADHD [12, 13]. Elhossiny et al. [12] conducted a randomized controlled trial using *Lactobacillus acidophilus* LB and reported significant improvements in attention and cognitive function when used as an adjunct to pharmacological treatment. Similarly, Ghanaatgar et al. [18] demonstrated that probiotic supplementation alongside methylphenidate led to enhanced behavioral outcomes compared to medication alone. A meta‐analysis by Liang et al. [19] further confirmed the therapeutic efficacy of probiotics in reducing core ADHD symptoms, emphasizing strain specificity and treatment duration as key factors influencing outcomes. These findings collectively reinforce the potential of probiotics as a safe and promising adjunctive strategy in managing neurodevelopmental disorders such as ADHD.

However, not all studies have reported consistent findings regarding the efficacy of probiotics in improving executive functions in children with ADHD. For example, Kumperscak et al. (2020) conducted a randomized controlled trial using *Lactobacillus rhamnosus* GG and found no significant improvement in core ADHD symptoms or executive functioning after 8 weeks of supplementation. Such discrepancies may stem from differences in strain specificity, dosage, and duration of intervention, as well as variability in outcome measures—with some studies focusing on behavioral symptoms rather than composite executive function scores like the GEC. Moreover, sample heterogeneity, including differences in age, baseline symptom severity, and comorbid conditions, may influence responsiveness to probiotic treatment. Cultural and dietary factors, such as habitual intake of fermented foods or fiber, can also modulate gut microbiota composition and potentially interact with probiotic efficacy [13]. These variations highlight the need for standardized protocols and strain‐specific investigations to better understand the therapeutic potential of probiotics in neurodevelopmental disorders.

These findings align with growing evidence supporting the gut–brain axis as a key mechanism affecting cognitive and behavioral consequences [11]. The present study specifically focused on probiotics including *Lactobacillus Acidophilus* , *Bifidobacterium Lactis* , and *Bifidobacterium Longum* , recognized for their capacity to modulate immune responses and diminish systemic inflammation [9, 20]. Improvements observed can be attributed to various mechanisms, including modulation of gut microbiota, enhancement of gut barrier integrity, and production of neuroactive compounds like gamma‐aminobutyric acid (GABA) and serotonin [10, 11]. These pathways are increasingly implicated in the pathophysiology of ADHD, suggesting that probiotics may exert their effects by addressing underlying biological dysregulations.

Moreover, the observed improvements in executive‐function‐related behaviors may be partly explained by the biological mechanisms of the probiotic strains used. *Lactobacillus acidophilus* has been shown to influence GABAergic signaling, which may positively affect emotional regulation and cognitive performance [21]. *Bifidobacterium longum* has been associated with enhanced stress resilience and cognitive function, potentially through modulation of tryptophan metabolism and cortisol levels [22]. Moreover, probiotics such as Lactobacillus and Bifidobacterium have demonstrated efficacy in managing pediatric neurodevelopmental disorders, including ADHD and autism spectrum disorder (ASD), by modulating gut microbiota composition. Animal studies further support these findings, showing that Bifidobacterium supplementation can improve memory and behavior in ADHD‐like models, with associated increases in neurotransmitters such as acetylcholine, dopamine, norepinephrine, and brain‐derived neurotrophic factor (BDNF), alongside reductions in glutamate levels [8].

Despite the promising outcomes, this study has some limitations. While fermented food intake in Iran is not as extensive or diverse as in East Asian cultures, certain staples such as yogurt, doogh, and traditional fermented dairy products are consumed regularly and may influence baseline gut microbiota. Regular consumption of fermented foods may influence baseline gut microbiota composition and potentially modulate responses to probiotic interventions. Also, the relatively short duration of the intervention and follow‐up periods may limit conclusions about long‐term clinical effects and sustainability. Furthermore, the study was conducted under stable pharmacological conditions, with no changes in methylphenidate dosage or behavioral interventions. While this design enhances internal validity, it may limit generalizability to settings with dynamic treatment adjustments. A key limitation of this study is that all outcome measures were based solely on parent‐reported BRIEF questionnaires. While parent reports provide valuable insights, relying exclusively on a single informant may introduce reporting bias and limit the generalizability of the findings. Future research should incorporate multi‐informant assessments, such as teacher reports and objective performance‐based cognitive tasks, to provide a more comprehensive and reliable evaluation of behavioral and cognitive outcomes. While the specific probiotic formulation used in this study (Bioflora) is commercially available in Iran, similar formulations containing *Lactobacillus acidophilus* , *Bifidobacterium lactis* , and *Bifidobacterium longum* are widely produced and distributed in various countries. These strains are commonly included in over‐the‐counter probiotic supplements globally, which enhances the reproducibility and potential clinical applicability of our findings across different settings.

Despite the limitations noted, the clinical implications of this study are noteworthy. Given the limitations and side effects of conventional ADHD treatments, probiotics may serve as a safe and promising adjunctive strategy. To enhance reproducibility and global applicability, future research should consider internationally standardized formulations and assess long‐term efficacy through extended interventions and follow‐up. Incorporating objective neuropsychological assessments and evaluating participants' dietary habits—particularly fermented food intake—will help clarify the true impact of probiotics on neurodevelopment. Further studies are also needed to optimize strain selection, dosage, and explore potential synergistic effects with existing therapies. Overall, these findings contribute to a growing body of evidence supporting biologically based, non‐pharmacological approaches to improve cognitive and behavioral outcomes in children with ADHD.

## 5 Conclusion

This study provides compelling evidence for the potential role of probiotic supplementation in enhancing executive functions in children with ADHD. The significant improvements observed in GEC scores among the intervention group highlight probiotics as a possible nonpharmacological adjunct to traditional ADHD treatments. These findings reinforce the growing understanding of the gut‐brain axis as a critical pathway influencing cognitive and behavioral outcomes.

Future studies should explore longer intervention periods, use objective neuropsychological assessments, and determine optimal probiotic strains and dosages. Additionally, integrating probiotics with existing ADHD treatments could provide a more comprehensive management approach. The findings emphasize the necessity for ongoing investigation into innovative, biologically based interventions, supporting the development of safer, more personalized strategies to improve cognitive and behavioral outcomes in children with ADHD.

## Author Contributions

S.D., A.P., P.S., Y.S., N.F., N.N., M.K., F.R., and M.G. designed the study and were involved in the data collection, analysis, and drafting of the manuscript. A.N., F.T., N.P.L., A.A., A.N., Y.S., P.B., and S.D. were involved in the design of the study, analysis of the data, and critically reviewed the manuscript. All authors read and approved the final manuscript.

## Funding

This work was supported by Guilan University of Medical Sciences, Rasht, Iran.

## Ethics Statement

This study was approved by the ethical committee of Guilan University of Medical Sciences, Rasht, Iran (Code: IR.GUMS.REC.1400.582) in accordance with the Declaration of Helsinki. Participants were given the necessary information about the purpose and duration of the study. The informed consent form clearly stated that participation in this study was completely voluntary and that participants could withdraw from the study at any time without giving any reason, without facing any consequences or losing access to benefits. Withdrawal from the study did not affect access to routine medical services. The treatment relationship with the center and physician remained unchanged.

## Consent

The authors have nothing to report (Code 3353).

## Conflicts of Interest

The authors declare no conflicts of interest.

## Data Availability

The data that support the findings of this study are available on request from the corresponding author. The data are not publicly available due to privacy or ethical restrictions.
