# Correction to “Probiotic Supplementation and Executive Function in Children With Attention‐Deficit/Hyperactivity Disorder”

**DOI:** 10.1002/npr2.70130

**Published:** 2026-05-24

A. Parhiz, P. Samani, M. Kamali, et al., “Probiotic Supplementation and Executive Function in Children With Attention‐Deficit/Hyperactivity Disorder,” Neuropsychopharmacology Reports 46, no. 1 (2026): e70084, https://doi.org/10.1002/npr2.70084.

There was a typographical error in the surname of the co‐author Ali Nori. The correct spelling should be “Nouri” instead of “Nori.”

The online version of this article has been corrected accordingly.

We apologize for this error.

